# Quantification of Craniofacial Growth Pattern Based on Deep Learning

**DOI:** 10.3390/bioengineering13030277

**Published:** 2026-02-27

**Authors:** Ziyi Hu, Yuyanran Zhang, Ningtao Liu, Xin Gao, Ziyu Huang, Guanglin Wu, Zhiyong Zhang, Shuang Wang

**Affiliations:** 1 Key Laboratory of Shaanxi Province for Craniofacial Precision Medicine Research, Department of Stomatology, College of Stomatology, Xi’an Jiaotong University, Xi’an 710004, China; 2 Department of Orthodontics, Affiliated Stomatological Hospital of Xi’an Jiaotong University, Xi’an 710004, China; 3 School of Computer, Luoyang Institate of Science and Technology, Luoyang 471023, China

**Keywords:** growth and development, lateral cephalometric radiographs, feature extraction, craniofacial bones, deep learning

## Abstract

**Background:** Childhood and adolescence constitute a critical period for craniofacial growth. Understanding its developmental patterns is essential for clinical decision-making in orthodontics and maxillofacial surgery. Traditional cephalometric analysis relies on manual landmarking, which oversimplifies complex morphology and introduces subjectivity. Although deep learning, a key artificial intelligence (AI) technology, has demonstrated remarkable performance in image analysis and classification, most methods still depend on manual annotations during training, perpetuating subjectivity and limiting model generalizability and robustness on large datasets. This hinders the development of objective, comprehensive methods to quantify craniofacial growth that account for its multi-tissue complexity. **Methods:** To address these limitations, this study developed an end-to-end deep learning framework based on lateral cephalometric radiographs from 41,625 individuals aged 4–18 years. Without relying on manual annotations, the model is designed to autonomously extract dynamic imaging features associated with continuous age intervals in craniofacial development and further discern features related to sexual dimorphism. Gradient-weighted Class Activation Mapping (Grad-CAM) was employed to visualize the learned features, generating population-averaged saliency maps that highlight age-related and sex-related patterns. Furthermore, we introduced two novel quantitative metrics, the Age-related Saliency Index (ASI) and the Sex-related Saliency Index (SSI), to evaluate the significance of developmental and dimorphic characteristics in key craniofacial regions. **Results:** Age-related saliency maps extended the focus from external contours to internal anatomical details of the bones, intuitively visualizing the relative importance of multiple bone regions during dynamic development, with the ASI providing a quantitative prioritization of these regions. The Sex-related Saliency Index (SSI) quantified the dynamic evolution of sexual dimorphism, demonstrating that early-stage differences were widely distributed across cranial bones and gradually became concentrated in the mandibular region by adulthood. **Conclusions:** This study established an end-to-end deep learning framework for analyzing large-scale lateral cephalometric radiographs. By generating age- and sex-related average saliency maps and their corresponding quantitative indices, we visualized and quantified the spatiotemporal growth dynamics and sexual dimorphism across distinct craniofacial skeletal regions throughout development. These findings not only validate established developmental theories but also provide novel insights into the coordinated growth patterns of craniofacial bones and sex-specific radiological characteristics, offering clinicians objective quantitative references for assessing developmental stages and guiding the timing of interventions targeting specific craniofacial regions.

## 1. Introduction

The human craniofacial complex undergoes a critical and intricate period of structural development across childhood and adolescence [1,2,3,4]. A profound understanding of its spatiotemporal growth patterns is essential for clinical decision-making regarding the timing and efficacy of interventions in orthodontics and maxillofacial surgery [5,6]. Conventionally, the quantitative assessment of these patterns has relied on landmark-dependent cephalometric analysis [7,8,9,10,11]. However, this methodology suffers from inherent limitations: it reduces complex morphology to sparse geometric abstractions based on manually identified points and fails to capture the heterogeneous growth within and between bones [12]; also, its reliance on subjective operator interpretation hinders the construction of objective, dynamic growth atlases that reflect population norms.

Deep learning, particularly Convolutional Neural Networks (CNNs) [13], has demonstrated substantial potential in dental and maxillofacial medical image analysis, with applications widely spanning caries detection [14], periodontal disease assessment [15], jaw lesion identification, and impacted tooth localization [16]. Although current mainstream supervised and semi-supervised learning approaches can achieve high diagnostic accuracy by leveraging large volumes of manually annotated data [17], they remain heavily reliant on the labor-intensive and potentially subjective annotation process conducted prior to training. This fundamental dependency does not overcome the deep-seated need for human prior knowledge inherent in traditional methods [18]. Consequently, it constrains the scalability and generalizability of these methods when applied to larger-scale clinical datasets. In the specialized area of craniofacial growth assessment, indirect staging methods based on cervical vertebrae or hand-wrist bones similarly face this limitation, failing to achieve holistic, continuous, and objective quantitative analysis of craniofacial skeletal developmental patterns [19,20,21].

Given these limitations, end-to-end deep learning offers a breakthrough approach. The strong correlation between growth and chronological age allows age to serve as a biological label encoding continuous developmental information [22]. By driving a model to perform the proxy task of high-precision age estimation, it autonomously learns the imaging features most representative of growth patterns [23,24]. Gender differences are also a critical factor in studying craniofacial developmental patterns [25]. Applying deep learning to gender classification tasks helps identify typical imaging feature differences between sexes at specific developmental stages. Meanwhile, visualization techniques can generate saliency maps, intuitively highlighting the key anatomical regions the model relies on for age or gender predictions [26]. This methodology has been validated in studies related to craniofacial aging patterns [24] and brain aging-related disease models [27]. Its adaptation to craniofacial development research holds the potential to provide a holistic, objective, and continuously dynamic perspective, quantifying spatiotemporal growth patterns and sex-related dimorphic features across developmental stages from a multi-tissue viewpoint.

Therefore, we hypothesized that an end-to-end deep learning framework could autonomously quantify spatiotemporal craniofacial growth patterns and sexual dimorphism without manual annotations. Based on this hypothesis, this study aims to construct an end-to-end deep learning framework to systematically investigate the craniofacial growth and development patterns in individuals aged 4 to 18 years. Considering the ethical guidelines and the typical level of cooperation attainable from children during clinical examinations, 4 years was selected as the lower age limit. The upper age limit was set at 18 years because the majority of craniofacial bones complete their growth by this age. The framework directly utilizes raw lateral cephalometric radiographs (LCRs) as input due to their standardized acquisition protocol, comprehensive anatomical coverage of the craniofacial region, and routine clinical availability [28,29,30,31,32].

In this study, the EfficientNet-B0 architecture was employed to execute learning tasks closely aligned with the extraction of growth and developmental features. Meanwhile, Grad-CAM was applied to generate age- and sex-related average saliency maps, which intuitively visualize the most essential imaging features learned from LCRs that characterize normative chronological growth patterns and sexual dimorphism. Furthermore, quantitative analysis was performed based on anatomical regions. Treating the craniofacial complex as a dynamic whole and without relying on manual annotations, this approach allowed us to rank and visualize the relative importance of different craniofacial regions across developmental stages, as well as their sex-related differences.

## 2. Materials and Methods

**Figure 1:**
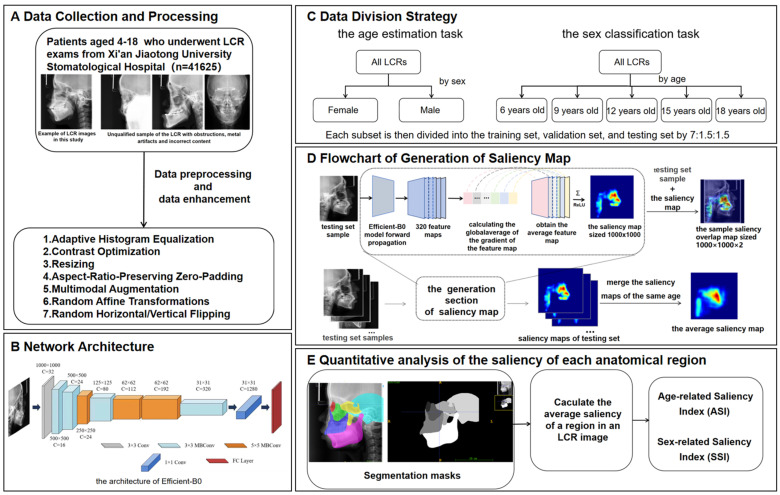
Overview of the methodology of this study. (**A**) Data collection and processing. (**B**) Network architecture. (**C**) Data division strategy. (**D**) Process of generating a saliency map. (**E**) Quantitative analysis of the saliency of each anatomical region. The saliency map represents the contribution of each region in the X-ray cephalic localization lateral image to the final age estimation result. Since it is generated on the basis of the trained EfficientNet-B0, the saliency map contains feature information for each age group. Each testing set sample is concatenated with its corresponding saliency map to generate a sample saliency overlap map of size 1000×1000×2, where 2 indicates the channel number. All the saliency maps of the same age are merged into the average saliency map corresponding to that age. The intensity of the red hue in the saliency map corresponds directly to the degree of age-related predictive importance within each anatomical region.

This study proposed an analytical framework for characterizing craniofacial growth patterns using age-related and sex-related saliency mapping on LCRs (Figure 1). The methodology comprised three modules: (1) training of deep learning models for concurrent age estimation and sex classification tasks; (2) generation of population-level mean saliency maps through aggregation of developmental features extracted by the trained models; (3) quantitative analysis of spatiotemporal saliency distributions at both pixelwise and instancewise scales through Age-related Saliency Index (ASI) and Sex-related Saliency Index (SSI).

### 2.1. Data Collection

In this study, 43,000 LCR images between the ages of 4 and 18 years were selected from the imaging database of Xi’an Jiaotong University Stomatological Hospital. The age range from 4 to 18 years was chosen in this study because it could basically cover the development period. Children under the age of 4 years are rarely examined with lateral radiographs, and X-ray examination cannot be performed on research subjects for research purposes due to medical ethical reasons, so we chose the age of 4 years as the lower limit of our study age range. This study was retrospective, and ethical approval had been obtained from the Xi’an Jiaotong University Stomatological Hospital (Ethical approval No. KY-QT-20240046), and the data had been anonymized, retaining only the age and sex labels. The age of each subject was calculated based on the date of birth documented in their official national identity card, which was used during patient registration. This was done by subtracting the imaging date from the date of birth, dividing by 365.25 (to account for leap years), and rounding to the nearest hundredth.

**Figure 2:**
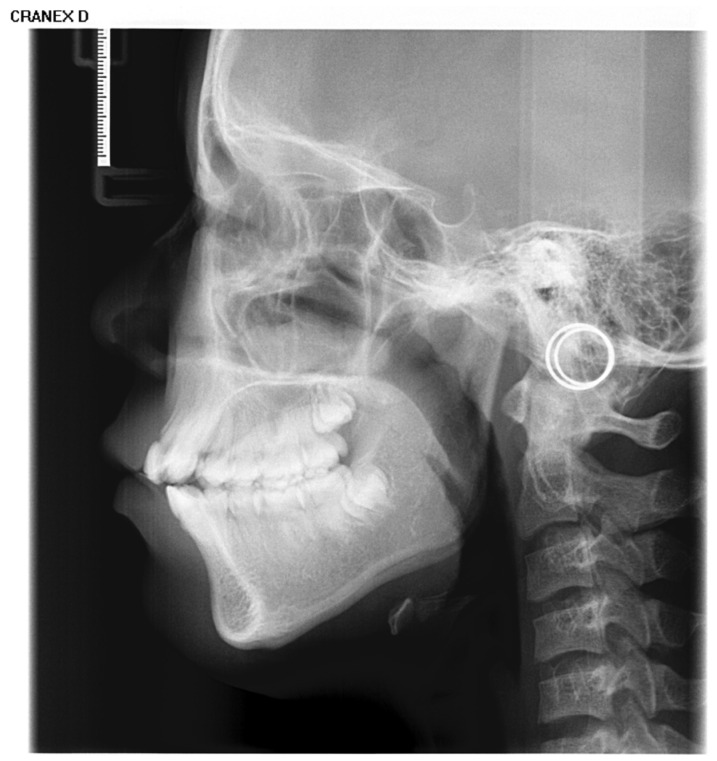
The LCR samples used in this study.

**Figure 3:**
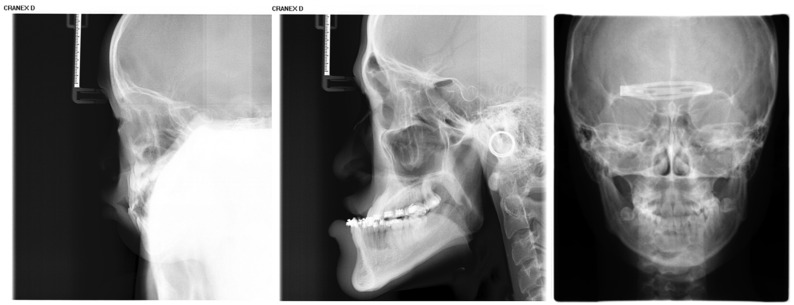
Illustration of ineligible lateral cephalometric radiograph samples included in this study, encompassing cases with occlusions, metallic artifacts, and content errors.

Inclusion criteria were as follows: (1) age between 4 and 18 years (up to and including 18.99 years); (2) absence of visible dental restorations in the oral cavity; (3) no substantial loss of craniofacial or dental tissues in the included samples. The LCR samples used in this study are shown in Figure 2. Exclusion criteria comprised (1) LCR images with improper positioning or incomplete anatomical coverage; (2) poor image quality resulting from blurring, underexposure, incorrect focal length, equipment malfunction, or the presence of obstructions such as metal artifacts; (3) documented history of head, facial, or neck tumors, or pathological conditions compromising bone or tooth integrity—including jaw tumors, cysts, osteomyelitis, jaw trauma, advanced periodontal disease, or tooth loss. The ineligible samples in this study are illustrated in Figure 3.

LCRs were acquired using a cephalostat for standardized head positioning. The Frankfort horizontal plane was aligned parallel to the floor via the machine’s ear rods and orbital pointer. The midsagittal plane was positioned parallel to the digital X-ray detector and perpendicular to the central X-ray beam. All images were obtained using a Cranex D digital X-ray unit (Soredex, Tuusula, Finland) with fixed exposure parameters: 73 kV, 7 mA, and an exposure time of 11.7 s. All LCRs were saved in the standard Digital Imaging and Communications in Medicine (DICOM) format.

Among the 43,000 LCRs, 1375 were eliminated based on the exclusion criteria. In summary, our dataset used for the age estimation and sex classification task included 41,625 patients, which contained 22,153 males and 19,472 females. A detailed description of the demographic characteristics of the patients who were prescribed LCR for each dataset is presented in Table 1.

### 2.2. Data Anonymization and Preprocessing

This study employed the DICOM Anonymizer tool to standardize the removal of all personally identifiable information from the medical images. This process ensured complete data anonymization, minimizing any risk to patient privacy, while retaining only age and sex as identifiable labels for analysis. Patient age at the time of imaging was accurately calculated using the birth date retrieved from the Hospital Information System (HIS) based on their unique identification number.

All LCRs were processed using the OpenCV library with a standardized preprocessing pipeline. First, adaptive histogram equalization was applied to optimize image contrast. Subsequently, to conform to a fixed input dimension while preserving the original anatomical proportions, each image was proportionally rescaled so that its longer side equaled 1000 pixels, followed by asymmetric zero-padding [33,34] on the shorter side to achieve a final 1000 × 1000 pixel canvas, keeping the craniofacial content centered. The training data underwent multimodal augmentation to improve model robustness, including random affine transformations (minor rotation, translation, and scaling) and random flipping. Furthermore, during the generation of group-averaged saliency maps, all individual saliency maps were algorithmically aligned to a consistent lateral orientation prior to averaging, ensuring the anatomical coherence and interpretability of the composite visualizations.

### 2.3. CNN Architecture

In this study, the EfficientNet-B0 architecture was selected as the core network due to its exceptional parameter efficiency and strong representational capacity. Compared to other convolutional neural networks, EfficientNet-B0 achieved better performance on the craniofacial growth classification task among the evaluated CNN models, making it particularly suitable as a baseline model for age estimation and a feature extractor for capturing craniofacial developmental patterns, as demonstrated in our prior benchmark study [24].

**Figure 4:**
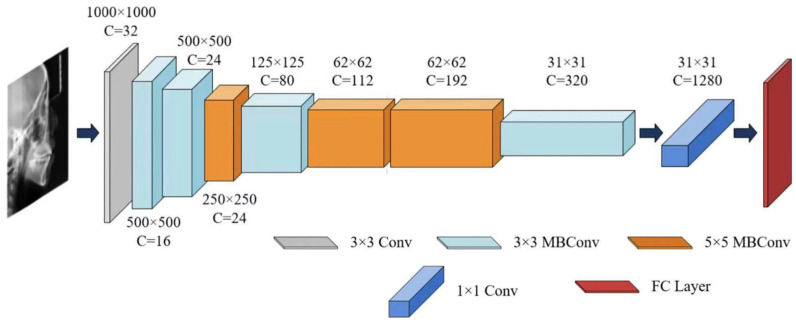
The architecture of EfficientNet-B0.

For the sex classification task, EfficientNet-B0 was trained to extract sex-discriminative imaging features from each training sample and compute probabilistic outputs based on these features [35,36], thereby characterizing patterns related to sexual dimorphism in growth and development. A summary of the EfficientNet-B0 architecture is provided in Table 2 and Figure 4.

### 2.4. Age Group Stratification

For the age estimation task, to avoid interference from inherent sexual dimorphism in craniofacial development, the dataset was first stratified by sex into male and female subsets. Each subset was then independently partitioned into training, validation, and testing sets using a ratio of 7:1.5:1.5. Age was modeled at one-year intervals to capture the continuous dynamics of growth and development (patient demographic characteristics are shown in Table 1).

For the sex classification task, the entire dataset was first divided into five age-specific subsets (ages 6, 9, 12, 15, and 18 years) to examine sex-related morphological differences during key growth windows. These points were selected based on their clinical and developmental significance in orthodontic monitoring and intervention (Table 3). The three-year assessment interval aligns with established practices in longitudinal growth studies [37,38], effectively capturing salient sexual dimorphism throughout puberty and adolescence while minimizing the impact of short-term variability. Subsequently, each age subset was partitioned into training, validation, and testing sets using the same 7:1.5:1.5 ratio.

### 2.5. Model Training

In this study, EfficientNet-B0 was trained to extract region-specific features associated with incremental age-related changes from each training sample and to perform age estimation based on these features. The model was trained using mini-batch gradient descent, where a batch of samples was fed into the network during each training iteration, and prediction errors were computed via a loss function. The Adam [53] optimizer was employed with a weight decay of 0.0001. Training was terminated for each network if no improvement in validation performance was observed for three consecutive epochs, or when the maximum predefined number of epochs (100) was reached. Upon completion of training, the network parameters that achieved the best performance on the validation set were selected and evaluated on the test set to obtain the final model performance as well as the fully trained EfficientNet-B0 model.

The L1 loss function was adopted for the age estimation task. The mean absolute error (MAE), a widely used evaluation metric in age estimation, was applied to assess model performance in this study. Given that age-related changes represent a continuous variable, deep learning-based cervical vertebral age estimation is formulated as a regression task, for which the L1 loss was utilized during training. The loss of the age estimation task $L_{a}$ is calculated as follows:

(1) $$L_{a}=\;\frac{1}{B}\sum_{n=1}^{B}\left|A^{n}-{\hat{A}}^{n}\right|$$

where ${\hat{A}}^{n}$ denotes the age estimation result corresponding to the n-th training sample of the training sample subset, $A^{n}$ denotes the age labeled by the age label corresponding to $A^{n}$, B is the batch size.

For further revealing patterns associated with sexual dimorphism in growth and development, EfficientNet-B0 was trained to extract sex-discriminative features from each training sample and compute sex classification probabilities based on these features. This task employed the cross-entropy loss function, widely used for classification problems. Since sex classification is a binary task, the Softmax function and cross-entropy loss function are used to calculate the cross-entropy loss value $L_{g}$ of sex classification probability and its corresponding sex label. Specially,

(2) $$p_{n}^{m}=\frac{e^{z_{n}^{m}}}{e^{z_{n}^{m}}+e^{z_{n}^{f}}}$$

(3) $$p_{n}^{f}=\frac{e^{z_{n}^{f}}}{e^{z_{n}^{m}}+e^{z_{n}^{f}}}$$

(4) $$L_{g}=\frac{1}{B}\sum_{n=1}^{B}\left[g_{n}\log(p_{n}^{m})+(1-g_{n})\log(p_{n}^{f})\right]$$

where $e^{z_{n}^{m}}$ and $e^{z_{n}^{f}}$ denote the output values of the n-th training sample of the subset of training samples corresponding to the sex of male and the sex of female, respectively, and $p_{n}^{m}$ and $p_{n}^{f}$ are the classification probabilities of male and female, respectively, and $g_{n}=1\;$ if the sex of the n-th training sample is male and $g_{n}=0$ if the sex of the n-th training sample is female;

In this study, the Adam optimizer was used for model training, with the learning rate adjusted via an exponential decay strategy—multiplied by a factor of 0.8 every 5 training epochs. Training was terminated if no improvement in validation performance was observed for three consecutive epochs or when the maximum predefined number of epochs (100 in this study) was reached. Upon completion of training, the model with the best performance on the validation set was selected for evaluation on the test set to obtain the final performance metrics.

### 2.6. Performance Metrics

All performance measures were calculated on the test set. This study employs multiple metrics for comprehensive model evaluation. For age estimation tasks, estimation accuracy is quantified through the MAE, root mean square error (RMSE), and coefficient of determination (R^2^). These metrics are defined as follows:

(5) $$MAE=\frac{1}{N}\sum_{n=1}^{N}\left|y_{n}-{y^{\prime }}_{n}\right|$$

(6) $$RMSE=\sqrt{\frac{1}{N}\sum_{n=1}^{N}{\left(y_{n}-{y^{\prime }}_{n}\right)}^{2}}$$

(7) $$R^{2}=1-\frac{\sum_{n=1}^{N}{\left(y_{n}-{y^{\prime }}_{n}\right)}^{2}\;}{\sum_{n=1}^{N}{\left(y_{n}-\bar{y}\right)}^{2}}\;\;\;\;\;\;$$

where N is the number of samples, the $\;y_{n}\;$ and ${y^{\prime }}_{n}\;$ are the age and predicted age of the n-th LCR image, respectively. $\bar{y}\;$ is the mean of the true ages.

For the sex classification task, the model generates continuous probability scores, where a value of 1 represents the female class (designated as the positive case) and 0 represents the male class (the negative case). Classification decisions are made by applying a decision threshold of 0.5 to these scores.

To ensure a comprehensive evaluation beyond overall accuracy, model performance was assessed using precision, recall, and the F1 score, all derived from the confusion matrix. The formulas for these metrics are defined as follows:

(8) $$Accuracy=\frac{TP+TN}{TP+TN+FP+FN}\;\;\;\;\;$$

(9) $$Precision=\frac{TP}{TP+FP}\;\;\;\;$$

(10) $$Recall=\frac{TP}{TP+FN}\;\;\;\;$$

(11) $$F1\;Score=\frac{Precision\times Recall}{Precision+Recall}\;\;\times 2\;$$

where TP represents the number of female cases correctly classified as female. TN represents the number of male cases correctly classified as male. FP represents the number of male cases incorrectly classified as female. FN represents the number of female cases incorrectly classified as male.

### 2.7. Generation of the Saliency Map

This study implemented gradient backpropagation through the Grad-CAM algorithm to characterize regional contributions within LCRs quantitatively for the identification of developmentally significant features.

First, each sample in the testing set is used as input to the trained $Effi_{best}$ to obtain the global average of the 320 feature maps for each sample, with the threshold of the region associated with the age estimation set to the 75th percentile of the average saliency. The formula for calculating the global average $\alpha_{r}$ of the gradient of the $r^{th}\left(1\leq r\leq 320,r\in Z\right)\;$ feature map $F^{r}$ is as follows:

(12) $$\alpha_{r}=\frac{1}{N_{p}}\sum_{i}\sum_{j}\frac{\partial \hat{A}}{\partial F_{ij}^{r}},$$

where $N_{p}$ denotes the number of pixel points of the feature map $M^{r}$; $F_{ij}^{r}$ denotes the pixel point at position (i,j) of ${\;F}_{ij}^{r};$ and $\hat{A}\;$ denotes the age estimation result of the input sample.

The weighted sum of all feature maps is computed via $\alpha_{k}$ as the weight to obtain the average feature map M:

(13) $$M=\sum_{k}\alpha_{k}M^{k};$$

To remove negative saliency, M was processed via the ReLU function to obtain the saliency map $M^{*}$ corresponding to this sample, as shown in Figure 1.

(14) M*=ReLU(M)

This study also conducted sex classification analysis on samples aged 6, 9, 12, 15, and 18 years. The Grad-CAM visualization framework was employed to both identify model-relevant salient regions and quantify age-specific sexual dimorphism patterns, with sex-discriminative features being extracted through systematic gradient analysis. For individual samples, saliency regions were determined by thresholding at the 75th percentile of globally averaged gradients across 320 convolutional feature maps. The final sex-related saliency maps were generated through a weighted linear combination of activated feature maps (Equation (13)) followed by rectified linear unit transformation (Equation (14)). All the saliency maps of the same sex are merged into the average sex-related saliency map, which reflects the general pattern of sex differences in the same age group.

### 2.8. Quantitative Analysis of the Saliency of Each Anatomical Region

**Figure 5:**
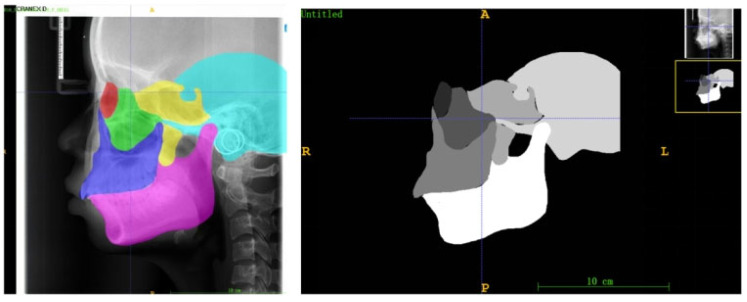
Examples of anatomical zone templates for an 18-year-old male, which contain delineations of the six craniofacial skeletal regions (red: socket; green: zygoma; blue: maxillae; yellow: sphenoid bone; blue: temporal bone; pink: mandible).

To quantitatively analyze the developmental saliency across the craniofacial complex, this study defined six core skeletal regions of interest (ROIs) on the lateral cephalometric radiographs (LCRs) based on their critical relevance in growth and development and their clinical significance: the orbital, zygomatic, maxillary, pterygoid, temporal, and mandibular regions (Figure 5). These regions were selected because they collectively cover the skeletal structures that undergo significant changes during craniofacial growth and development, and are representative of understanding growth chronology, spatial coordination, and the expression of sexual dimorphism.

The selection and delineation of these regions were based on their identifiable boundaries formed by established cephalometric landmarks and characteristic radiographic anatomy:

Orbital region: The quadrilateral radiolucent area bounded by the superior and inferior orbital rims and the lateral orbital margin.

Zygomatic region: The dense, anteriorly convex radiopacity corresponding to the zygomatic body, anteriorly connected to the zygomaticofrontal and zygomaticomaxillary sutures, and posteriorly to the zygomatic arch.

Maxillary region: The quadrilateral radiopaque area comprising the maxillary body, extending from the infraorbital rim superiorly to the alveolar process inferiorly, with the posterior boundary at the pterygomaxillary fissure, and containing the maxillary sinus radiolucency.

Sphenoid region: Includes the sella turcica, dorsum sellae, and clinoid processes; the pterygoid plates located posterior-superior to the maxillary tuberosity; and the pneumatized sphenoid sinus.

Temporal region: Primarily the squamous part, forming the curved bony plate of the lateral wall of the middle cranial fossa, bounded superiorly by the temporal line and with the glenoid fossa anteroinferiorly.

Mandibular region: The continuous, horseshoe-shaped radiopacity encompasses the mandibular body, ramus, condyle (within the glenoid fossa), and coronoid process.

To ensure comparability of quantitative analyses across all subjects, age- and sex-specific anatomical templates were constructed for each ROI. First, 20 LCR images were randomly selected for each year of age and for each sex from the overall dataset. An experienced orthodontist manually segmented the six ROIs on every image in this subset using ITK-SNAP software (Insight Toolkit - Snake Automatic Partitioning, Version 3.8; University of Pennsylvania, USA). The segmentation results from these 20 LCRs for each age-sex group were then averaged to generate a consensus mask for each ROI (Figure 5).

Subsequently, for quantitative analysis, the regional saliency for each subject was derived through an ensemble process to enhance robustness and representativeness. Each subject’s corresponding sex- and age-specific developmental saliency map was quantitatively analyzed against all 20 individual segmentations from its matching age-sex group. Specifically, for a given ROI, the mean saliency intensity was calculated separately within the boundaries of each of the 20 segmentation masks. The final ASI or SSI value for that subject and ROI was then computed as the average of these 20 individual measurements.

After visualizing age-related changes in bony and dental tissues through developmental saliency maps derived from LCR images, this study aimed to quantify regional growth patterns. Saliency within each anatomical region was measured by computing the mean pixel intensity relative to the region area. The Age-related Saliency Index (ASI) for a given region $C_{i}$ at age a is defined as:

(15) $${\mathrm{ASI}}_{C_{i}}^{a}=\frac{1}{N_{s}}\sum_{(i,j)\in M_{C_{i}}}S_{ij}^{a}\cdot 1(S_{ij}^{a})$$

where $M_{C_{i}}$ denotes the set of pixel coordinates within the region, $S_{ij}^{a}$ is the saliency intensity at the location $\left(i,j\right)$ from the age estimation task, and $N_{s}$ is the number of pixels with saliency above a threshold t. The indicator function is applied to exclude low-saliency areas:

(16) $$1(S_{ij}^{a})=\left\{\begin{array}{cc} 1, & S_{ij}^{a}\geq t\\ 0, & S_{ij}^{a}<t \end{array}\right.$$

Here, t is set to the 75th percentile of the age-specific saliency distribution across the sample. This threshold focuses the analysis on regions that contribute meaningfully to age-related morphological changes.

To systematically quantify spatial patterns of sexual dimorphism, we propose the Sex-related Saliency Index (SSI). While its computational form aligns with that of the ASI, the SSI is derived from saliency maps generated by the sex classification task, reflecting features that distinguish between males and females. For an anatomical region $C_{i}$ at age a, the SSI is calculated as:

(17) $${\mathrm{SSI}}_{C_{i}}^{a}=\frac{1}{N_{s}}\sum_{(i,j)\in M_{C_{i}}}S_{ij}^{a}\cdot 1(S_{ij}^{a})$$

Here, $S_{ij}^{a}$ corresponds to saliency intensities from the sex-classification output, and the same thresholding procedure (Equation (16)) is applied, with t set to the 75th percentile of sex-related saliency across the dataset. This approach ensures that the SSI captures anatomically meaningful regions indicative of sex-specific morphological variation.

Both ASI and SSI adopt the same quantitative framework to measure regional importance—normalized mean saliency above a defined threshold. The key distinction lies in the source of the saliency maps: ASI is based on features relevant to age progression, whereas SSI is derived from features discriminative of sex differences. They provide complementary perspectives on craniofacial development within a unified analytical scheme.

## 3. Results

### 3.1. Model Performance Evaluation

The EfficientNet-B0 model demonstrates great performance in age estimation (Table 4). The model achieved high predictive accuracy, with an overall mean absolute error (MAE) of 0.6447 years and a root mean square error (RMSE) of 0.8578. The coefficient of determination (R^2^) exceeded 0.93, collectively demonstrating strong predictive stability and explanatory power. This robust predictive performance confirms that the model successfully learned reliable and highly informative imaging features that are strongly correlated with craniofacial growth. These features form a credible foundation for the subsequent generation of saliency maps and the calculation of the ASI.

For sex classification, the model achieved exceptional and highly consistent performance across all five age groups (Table 5), with accuracy, precision, recall, and F1 scores consistently exceeding 99.5%. This robust and stable performance across development—exemplified by perfect precision (100%) in the 6-, 9-, and 12-year-old cohorts and perfect recall (100%) in the 18-year-old cohort—validates the model’s reliability. This high level of consistency provides a solid and dependable foundation for the subsequent steps: visualizing sex-discriminative features via saliency maps, constructing the SSI, and conducting a quantitative spatial analysis of craniofacial sexual dimorphism.

### 3.2. Visualization and Quantitative Analysis of Characteristics Related to Growth

**Figure 6:**
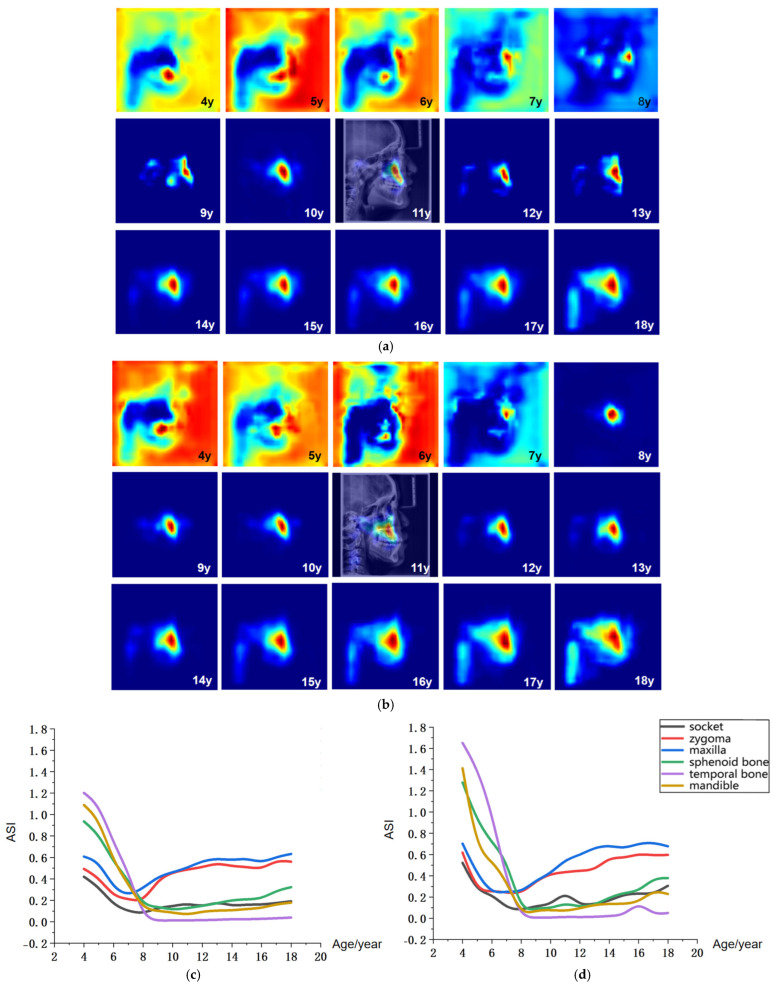
Visualization of age-related saliency features. (**a**) Population-averaged age-related saliency maps for females aged 4–18 years; (**b**) Corresponding saliency maps for males. In (**a**,**b**), saliency is mapped on a blue-to-red gradient, with red indicating a higher Age-related Saliency Index (ASI). A representative saliency map from an 11-year-old subject is overlaid on the original LCR to illustrate anatomical correspondence. (**c**) Female ASI values across anatomical structures and ages. (**d**) Male ASI values across anatomical structures and ages.

Figure 6a,b presents the age-related average saliency maps for females aged 4 to 18 years, illustrating the distribution of developmental saliency across craniofacial regions in LCR images. To clearly demonstrate the anatomical correspondence of these salient regions, the saliency map of a representative 11-year-old subject is overlaid onto their original LCR image, showing the precise spatial alignment between salient areas and anatomical structures. For visualization, the saliency intensity is mapped to a color gradient from blue to red, where increasingly red hues indicate higher age-related saliency in the corresponding craniofacial region.

**Figure 7:**
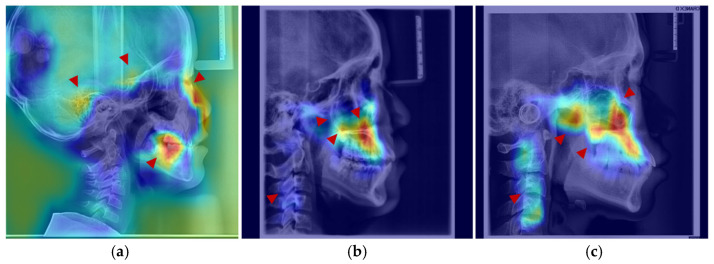
Anatomical structures exhibiting developmental saliency in average age-related saliency maps. (**a**) A 6-year-old female example (LCR with overlaid saliency map). Arrows indicate salient regions during the 4–7-year period: temporal bone, sphenoid region, orbit, and dentition. (**b**) A 12-year-old female example. Arrows highlight salient regions in the 8–12-year period: maxilla (tuberosity and palatine bone), zygomatic bone, pterygoid process, and cervical vertebrae. (**c**) An 18-year-old male example. Arrows point to salient regions in the 13–18-year period, including the maxilla, mandible, zygomatic bone, and cervical vertebrae.

The age-related saliency maps (Figure 6) revealed that during early development (ages 4–7 years), the sphenoid, temporal bones, and dentition exhibited high saliency (Figure 7a). At this stage, the outer background of the craniofacial contour in LCR images also showed relatively strong saliency. Quantitative analysis of the Age-related Saliency Index (ASI) for each anatomical structure (Figure 6 and Table 6 and Table 7) indicated that the saliency of both the temporal and sphenoid bones declined between ages 4 and 7. After age 8, the ASI for the maxilla and zygoma showed a synchronous, rapid increase (Figure 7b), whereas the ASI for the temporal bone remained at a low baseline without significant elevation. High saliency was observed in the mandible, as well as in internal anatomical regions such as the maxillary tuberosity and palatine process within the maxilla (Figure 7b,c), and the pterygoid process of the sphenoid bone.

After age 10, salient regions expanded to include the zygomatic bone, mandibular ramus, and cervical vertebrae (Figure 7b,c). During this period, the ASI of the temporal bone remained low without a notable increase, while the mandible, pterygoid region, and orbits exhibited a gradual rise in saliency—a trend slightly more pronounced in males than in females, consistent with the patterns observed in the average saliency maps. By age 18, saliency had increased across most craniofacial areas, with further expansion in the cervical region (Figure 7c). In late adolescence, males demonstrated higher saliency in the mandibular and zygomatic regions.

**Figure 8:**
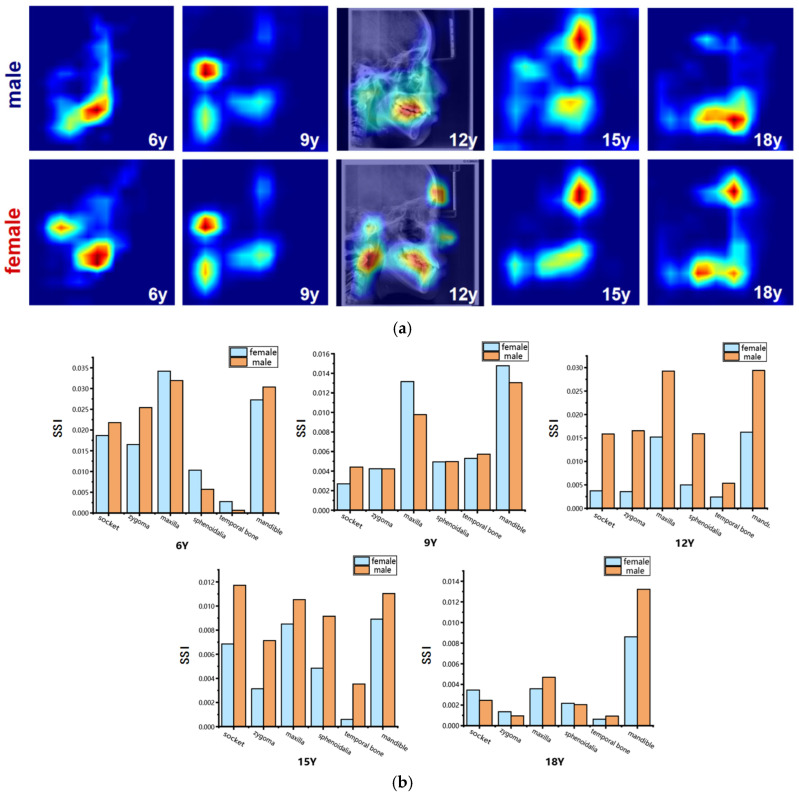
Visualization of sex-related saliency features. (**a**) Sex-related saliency maps for males and females at ages 6, 9, 12, 15, and 18. Saliency is represented on a blue-to-red gradient, with red indicating higher SSI. For anatomical reference, the saliency map of a representative 12-year-old subject is overlaid on the original LCR image. (**b**) Comparison of Sex-related Saliency Index (SSI) values between males and females across anatomical structures at the five selected ages.

To visualize the intensity of sexual dimorphism across craniofacial skeletal regions, average sex-related saliency maps were generated. These maps intuitively highlight key regions of sex difference and allow observation of their distribution patterns across developmental stages, providing a visual foundation for understanding the anatomical basis of craniofacial sexual dimorphism. In this visualization, SSI values are mapped onto a continuous blue-to-red color gradient: red indicates higher SSI values, representing anatomical structures with greater contribution to sex classification and stronger sexual dimorphism; blue denotes lower sex-discriminative relevance. A representative SSI map from a 12-year-old subject is overlaid on the corresponding LCR image for anatomical reference (Figure 8).

The results of the sex-specific pattern shown in Figure 8, we found that at 6 years of age, males’ significant areas were concentrated in the mandible, especially in the dentition region, whereas among females it showed to be mainly in the mandibular body as well as in the condylar region; at the age of 9 years, both sexes showed significant developmental features in the forehead, mandibular body, and cervical vertebrae regions. Nevertheless, females had a larger range of significant areas in the mandibular and cervical vertebrae regions compared to the males; at 12 years of age, the entire maxillofacial region was conspicuous in males, whereas in females it was concentrated on specific development of the mandible, and the soft tissues of the maxillary nasal region; at 15 years of age, males had stronger forehead, orbital, and maxillomandibular conspicuity than females; and at 18 years of age, the mandible continued to maintain a high level of conspicuousness in males, whereas the conspicuous regions in females gradually concentrated toward the chin and mandibular angle regions. Quantitative results (Figure 8 and Table 8 and Table 9) showed that at age of 6 years, the maxilla and mandible were the main sex-related regions, with higher SSI in the temporal, pterygoid, and mandible in males and more significant in the maxilla, orbit, and zygomatic bone in females; at age 9 years, the mandible showed to be the most significantly different region, with a higher SSI among the females; at the age of 12 and 15 years, the SSI of the males were significantly higher than that of the females for all skeletal regions, especially for the socket. By the time they reached adulthood at age 18 years, the mandible was the most significantly different region, which was significantly higher among the males compared to the females. In contrast, the differences in the other regions were significantly weaker.

## 4. Discussion

This study proposes an end-to-end deep learning framework for the systematic quantification of craniofacial growth patterns and sexual dimorphism from lateral cephalometric radiographs (LCRs). Traditional methods reliant on manual feature extraction and analysis suffer from inherent limitations in efficiency and objectivity, struggling to comprehensively process the high-dimensional and complex detailed information within medical images. To overcome this bottleneck, this study employs deep learning technology centered on convolutional neural networks. By directly processing raw images without any manual annotation and utilizing age estimation and sex classification as proxy tasks, the framework drives the model to autonomously learn the most discriminative morphology features related to development in an end-to-end manner. The model based on EfficientNet-B0 achieved excellent performance: a mean absolute error of 0.6447 years for age estimation and an accuracy exceeding 99.55% for sex classification. These results validate the reliability of the learned features and establish a solid foundation for subsequent analysis of growth patterns and research into model interpretability.

LCRs were selected as the primary imaging modality due to their comprehensive coverage of craniofacial bones and cervical vertebrae, which offer richer developmental features compared to periapical or panoramic radiographs. Utilizing a large-scale LCR dataset, an end-to-end, fully automated deep learning framework was employed for age-related feature extraction. Grad-CAM was applied to generate population-level average age-related saliency maps. Furthermore, an age-related saliency index was proposed to achieve a quantitative ranking of developmental features across different craniofacial regions. This method provides an objective and dynamic assessment tool for clinical practice. It not only validates established developmental patterns but also reveals localized growth characteristics difficult to capture with traditional measurements, thereby offering new insights for determining the timing and target regions of clinical interventions.

The systematic analysis revealed age-related patterns in craniofacial growth and development. Craniofacial development involves complex changes in both bone and soft tissue. During the early stage (4–7 years), growth is dominated by the cranial region, with a rapid increase in cranial volume—approximately 90% of cranial growth is completed by age [54,55]. The high saliency observed in regions such as the sphenoid and temporal bones on the saliency maps corresponds to the substantial overall dimensional changes in thechanges of the craniofacial skeleton during this phase, consistent that the cranial region matures earlier than the facial skeleton [56,57]. After age 8, age-related saliency shifts from the entire cranium to more localized regions, with the maxilla showing the most prominent age-related saliency. This transition aligns with the evolving growth pattern during adolescence: although the maxilla exhibits a smaller absolute growth increment compared to the mandible, it undergoes significant relative morphological remodeling during puberty. Moreover, its growth—primarily through sutural activity and deposition at the maxillary tuberosity—is more temporally consistent and exhibits less individual variation [1,58,59]. Consequently, it demonstrates higher predictive saliency in the age estimation model. This finding suggests that the model captures stable and predictable associations between morphological changes and age, rather than merely the magnitude of growth. Thus, the greater inter-individual variability in mandibular morphology may reduce its weight in age estimation, leading the model to prioritize the more consistent maxillary features. This finding not only elucidates the heterogeneity of pubertal growth but also positions maxillary morphology as a stable imaging biomarker, offering a quantitative basis for timing maxillary orthopedic interventions in clinical practice.

Furthermore, the saliency maps visualized coordinated growth patterns and internal structures that are challenging to assess through traditional methods. The synchronous increase in saliency of the maxilla and zygoma after age 8 underscores their functional unit in maintaining midfacial height, thereby clarifying the biological basis of the maxillo-zygomatic complex as a coordinated developmental unit from a clinical perspective. Equally important was the identification of high saliency in the pterygoid processes of the sphenoid bone, a region notoriously difficult to measure with conventional cephalometry. Its pronounced saliency highlights its dual role in supporting posterior maxillary positioning and influencing nasopharyngeal airway dimensions [60,61], warranting greater clinical attention. Additionally, by localizing changes in internal structures via saliency mapping, this study revealed persistently high age-related saliency in the maxillary tuberosity and palatine process regions from ages 9 to 18, providing quantitative support for existing theories on long-term dental arch morphological changes [44,62]. These findings not only explain the effectiveness of rapid maxillary expansion during early puberty from a developmental dynamics perspective but also suggest that these high-saliency regions may serve as imaging biomarkers for assessing individual skeletal responsiveness potential.

Sexual dimorphism during craniofacial skeletal growth and development represents significant characteristics [63,64,65]. In this study, an automated quantitative analysis of coordinated multi-bone sexual patterns across the craniofacial complex was achieved using an EfficientNet-B0-based deep learning framework, combined with Grad-CAM to generate sex-related saliency maps and the SSI. The findings reveal a clear stage-wise evolution of sexual dimorphism (Figure 8): differences were widely distributed across the jaws and partial cranial regions in early childhood (age 6); they gradually concentrated in the functional areas of the maxilla and mandible during adolescence (ages 9–15), concurrently reflected in the contours of facial soft tissues; and by adulthood (age 18), they became distinctly focused on the mandible, with males exhibiting higher SSI values. These results not only confirm the central role of the mandible in sexual expression but also indicate that structures such as the maxilla and zygoma are already involved in sexual differentiation during early developmental stages. This quantitative framework provides a reference basis for conducting sex-sensitive clinical assessments and formulating individualized orthodontic and orthognathic treatment plans.

In addition, the age estimation model constructed in this study, by analyzing complete LCRs, enables the assessment of an individual’s developmental status relative to their peers and provides a quantitative basis for clinically evaluating potential developmental abnormalities. The results indicate that the feature saliency of the cervical vertebral region in age estimation remains consistently lower than that of the craniofacial skeletal regions. This observation aligns with previous findings suggesting that cervical vertebral maturation staging may lag in predicting early growth peaks [40], implying that craniofacial bones may more sensitively reflect individual developmental progression. Consequently, this approach offers a more precise reference for determining growth stages in orthodontic and maxillofacial treatment planning. By employing an end-to-end deep learning framework, this study establishes an objective analytical pathway from raw imaging data to the intuitive quantification of craniofacial developmental patterns, thereby advancing craniofacial growth assessment from traditional manual landmark- and indirect indicator-based methods toward an evolved paradigm centered on data-driven, holistic analysis.

Nevertheless, several limitations of this study should be acknowledged. First, the model was developed and validated on a retrospective, single-center dataset, which may constrain its generalizability to populations with differing genetic backgrounds, environmental exposures, or radiographic acquisition protocols. Second, reliance on two-dimensional lateral cephalometric radiographs inherently limits the assessment of transverse craniofacial dimensions and introduces projective superimposition of structures, potentially obscuring important three-dimensional morphological details. Third, while Grad-CAM effectively highlights image regions influential for the model’s predictions, the specific anatomical or radiological correlates of these high-saliency features—such as localized bone density, trabecular architecture, or subtle contour changes—remain unclear, and their underlying biological mechanisms require deeper investigation.

To address these limitations, future research should focus on (1) validating and refining the model using multi-center, multi-ethnic cohorts to improve robustness and clinical applicability; (2) aiming to transcend the dimensional limitations of conventional radiographs by integrating three-dimensional imaging. The pioneering work of Olszewski et al. established the theoretical and practical framework for 3D cephalometric analysis using CT data, demonstrating its superiority in providing comprehensive spatial information without structural superimposition [66]. Building upon this foundation and leveraging the clinical accessibility of Cone-Beam Computed Tomography (CBCT), future research can apply similar end-to-end deep learning frameworks to 3D volumes. This would unlock a truly holistic quantification of growth, allowing for the analysis of transverse dimensions, bilateral asymmetries, and complex surface morphologies. Furthermore, the development of anatomically specific 3D reference systems, such as those for the mandible [67], provides a robust methodological template for conducting precise, region-focused analyses in three dimensions, akin to the regional ASI/SSI indices proposed in our current 2D study; and (3) correlating deep-learning-derived saliency features with hand-crafted radiomic features or biomechanical parameters to enhance interpretability and foster a more mechanistic understanding of craniofacial growth patterns.

In summary, this study presents the end-to-end deep learning framework for analyzing craniofacial developmental patterns. By directly processing raw radiographs, the model autonomously extracts intrinsic imaging features related to age and sex. Integrated with Grad-CAM, we generated population-averaged saliency maps and proposed the ASI and SSI indices, enabling intuitive visualization and quantitative ranking of regional developmental importance and sexual dimorphism, thereby overcoming the inherent limitations of traditional landmark-dependent analysis. The framework establishes a systematic, dynamic, and quantifiable assessment paradigm, offering clinicians objective and quantitative references for evaluating developmental stages and guiding intervention timing in orthodontics and dentofacial orthopedics.

## Figures and Tables

**Table 1:** The age and sex distribution of the LCR images.

	Male				Female			
Age Group (Year)	Train	Val	Test	All	Train	Val	Test	All
4–5	574	117	131	822	385	72	77	534
5–6	391	63	71	525	332	70	55	457
6–7	428	78	104	610	424	94	100	618
7–8	698	189	141	1028	675	146	133	954
8–9	1589	333	335	2257	946	204	198	1348
9–10	1540	332	330	2202	938	198	212	1348
10–11	1584	354	294	2232	940	205	245	1390
11–12	1144	235	253	1632	1134	279	245	1658
12–13	1514	335	341	2190	1725	366	419	2510
13–14	1250	249	252	1751	1327	296	312	1935
14–15	1386	296	238	1920	1239	285	258	1782
15–16	1314	290	266	1870	1087	285	256	1628
16–17	762	167	164	1093	953	194	195	1342
17–18	632	133	124	889	638	123	134	895
18–19	794	161	177	1132	750	153	170	1073
all	15,600	3332	3221	22,153	13,493	2970	3009	19,472

**Table 2:** EfficientNet-B0 network architecture.

Stage	Operator	Resolution	Channel	Layers
I	${\hat{F}}_{i}$	${\hat{W}}_{i}$ × ${\hat{H}}_{i}$	${\hat{C}}_{i}$	${\hat{L}}_{i}$
1	Conv3 × 3	1000 × 1000	32	1
2	MBConv1, k3 × 3	500 × 500	16	1
3	MBConv6, k3 × 3	500 × 500	24	2
4	MBConv6, k5 × 5	250 × 250	24	2
5	MBConv6, k3 × 3	125 × 125	80	3
6	MBConv6, k5 × 5	62 × 62	112	3
7	MBConv6, k5 × 5	62 × 62	192	4
8	MBConv6, k3 × 3	31 × 31	320	1
9	Conv1 × 1 & Pooling & FC	31 × 31	1280	1

**Table 3:** Summary of developmental characteristics and clinical significance at key age points in craniofacial growth and development.

Age (Years)	Developmental Stage and Clinical Characteristics	Clinical Significance and Considerations
6	Transition to mixed dentition begins; eruption of first permanent molars establishes occlusal height. Craniofacial growth shifts from the rapid infantile pattern to a dynamic remodeling phase [38].	Marks the beginning of permanent dentition establishment and serves as a critical baseline age for formulating long-term treatment plans. Early interceptive treatment can be initiated.
9	Pre-peak growth phase before adolescent growth spurt; midpalatal suture not fully ossified; mandibular growth potential remains high. Represents the optimal window for orthopedic treatments such as rapid maxillary expansion (RME) and maxillary protraction (MP) [39].	Allows full utilization of residual growth potential for skeletal modification to improve maxillary and zygomatic width and harmonize jaw relationships.
12	Most children enter the peak of their adolescent growth spurt. Facial growth surge slightly lags behind stature spurt; mandibular length growth reaches its peak; sex differences in growth rate and direction become particularly pronounced [40].	Represents the peak growth phase and a critical period for functional orthopedic treatment. Close attention should be paid to sex- and timing-related variations in the growth spurt.
15	Late adolescence with decelerated but ongoing growth [41]. Zygomatic width growth is largely complete [42,43], while areas such as the maxillary tuberosity continue active remodeling [44].	Important for evaluating treatment stability and deciding whether to await completion of residual growth [45,46]. Continued micro-growth in regions like the maxillary tuberosity should be monitored.
18	Craniofacial skeletal morphology is largely stabilized [47,48,49]. Skeletal sexual dimorphism is fully expressed: males exhibit larger skeletal dimensions with more angular contours; females show softer and smoother outlines [50,51,52].	The focus of growth assessment shifts from prediction to confirmation of maturity. Skeletal sex differences inform aesthetic evaluation and final planning for combined orthodontic-orthognathic treatment.

**Table 4:** Model performance for the age estimation task.

Sex	Index of Model Performance		
	Mean Absolute Error (MAE)	Root Mean Square Error (RMSE)	Coefficient of Determination (R^2^)
Male	0.5588	0.7549	0.9565
Female	0.7299	0.9488	0.9322
All	0.6447	0.8578	0.9444

**Table 5:** Model performance of sex classification tasks for different age groups.

Age (Year)	Index of Model Performance			
	Accuracy	Precision	Recall	F1 Score
6	0.9977	1.0000	0.9973	0.9987
9	0.9955	1.0000	0.9990	0.9995
12	0.9986	1.0000	0.9982	0.9991
15	0.9986	0.9991	0.9991	0.9991
18	0.9992	0.9987	1.0000	0.9993

**Table 6:** Age-related Saliency Index (ASI) Values for Growth and Development of Anatomical Structures in Male Subjects Aged 4–18 Years.

Age (Year)	Region					
	Socket	Zygoma	Maxilla	Sphenoid Bone	Temporal Bone	Mandible
4	0.5228	0.6182	0.7025	1.2783	1.6504	1.4122
5	0.2532	0.2530	0.4136	0.9151	1.4121	0.6416
6	0.2274	0.2582	0.2308	0.7151	0.9874	0.5412
7	0.0959	0.2426	0.2542	0.5518	0.3489	0.3485
8	0.0759	0.2304	0.2462	0.0591	0.0119	0.0284
9	0.1177	0.3520	0.3576	0.0974	0.0056	0.0746
10	0.1259	0.4192	0.4212	0.0913	0.0063	0.0789
11	0.2550	0.4342	0.5532	0.1445	0.0133	0.0680
12	0.1189	0.4483	0.5954	0.1056	0.0112	0.0935
13	0.1359	0.4526	0.6665	0.1247	0.0133	0.1275
14	0.1628	0.5738	0.6861	0.1957	0.0218	0.1370
15	0.2255	0.5665	0.6557	0.2411	0.0289	0.1350
16	0.2342	0.6076	0.7042	0.2526	0.1526	0.1526
17	0.2273	0.5924	0.7138	0.3766	0.0352	0.2621
18	0.3058	0.5971	0.6778	0.3790	0.0498	0.2289

**Table 7:** Age-related Saliency Index (ASI) Values for Growth and Development of Anatomical Structures in Female Subjects Aged 4–18 Years.

Age (Year)	Region					
	Socket	Zygoma	Maxilla	Sphenoid Bone	Temporal Bone	Mandible
4	0.4203	0.4933	0.6081	0.9358	1.2018	1.0888
5	0.3242	0.4119	0.5623	0.8162	1.0925	0.9606
6	0.1575	0.2351	0.3121	0.5543	0.7320	0.5588
7	0.0989	0.2134	0.2435	0.3914	0.4669	0.3504
8	0.0753	0.1825	0.3121	0.1504	0.0427	0.1214
9	0.1330	0.3947	0.4220	0.1402	0.0077	0.1013
10	0.1443	0.4611	0.4616	0.1100	0.0146	0.0869
11	0.1728	0.4886	0.5040	0.1283	0.0127	0.0642
12	0.1385	0.5081	0.5676	0.1482	0.0158	0.0958
13	0.1880	0.5487	0.5894	0.1721	0.0181	0.1052
14	0.1466	0.5178	0.5739	0.2023	0.0244	0.1059
15	0.1619	0.5123	0.5924	0.2089	0.0238	0.1194
16	0.1594	0.4895	0.5497	0.2146	0.0276	0.1277
17	0.1747	0.5743	0.6045	0.2902	0.0326	0.1642
18	0.1911	0.5605	0.6324	0.3227	0.0395	0.1780

**Table 8:** Sex-related Saliency Index (SSI) Values for Growth and Development of Anatomical Structures in Male Subjects at ages of 6, 9, 12, 15, and 18 Years.

Age (Year)	Region					
	Socket	Zygoma	Maxilla	Sphenoid Bone	Temporal Bone	Mandible
6	0.0218	0.0254	0.0319	0.0057	0.0006	0.0304
9	0.0044	0.0042	0.0098	0.0050	0.0057	0.0130
12	0.0159	0.0166	0.0293	0.0159	0.0053	0.0294
15	0.0117	0.0071	0.0105	0.0091	0.0035	0.0110
18	0.0025	0.0009	0.0047	0.0020	0.0009	0.0132

**Table 9:** Sex-related Saliency Index (SSI) Values for Anatomical Development in Female Subjects at Age of 6, 9, 12, 15, and 18 Years.

Age(Year)	Region					
	Socket	Zygoma	Maxilla	Sphenoid Bone	Temporal Bone	Mandible
6	0.0187	0.0165	0.0342	0.0103	0.0027	0.0273
9	0.0027	0.0042	0.0132	0.0049	0.0053	0.0148
12	0.0037	0.0036	0.0152	0.0050	0.0024	0.0162
15	0.0069	0.0031	0.0085	0.0048	0.0006	0.0089
18	0.0035	0.0013	0.0036	0.0022	0.0006	0.0086

## Data Availability

The data used in this study are not open access due to privacy and security concerns. After the sharing agreement is obtained, it can be shared with third parties for reasonable use, and relevant requests should be addressed to Z.Z. (zzy20011126@mail.xjtu.edu.cn). To enable a complete run of the code shared in this study, a minimum amount of desensitized sample data is shared with the code. The source code of this study is provided at https://github.com/hzy739090555/ASI-SSI/tree/main (accessed on 1 October 2025).
